# No obvious effect on mortality from a patient choice reform expanding access to opioid disorder treatment – results from a natural experiment of policy change in Sweden

**DOI:** 10.1186/s13011-023-00577-4

**Published:** 2023-11-06

**Authors:** Anders Håkansson, Sahar Janfada-Baloo, Jonas Berge

**Affiliations:** 1 Faculty of Medicine, Department of Clinical Sciences Lund, Lund University, Psychiatry, Lund, Sweden; 2 Region Skåne, Malmö Addiction Center/Competence Center Addiction, Malmö, Sweden

**Keywords:** Overdose, Mortality, Opioid use disorders, Opioid maintenance treatment

## Abstract

**Background:**

Opioid-related overdose deaths remain a common cause of death in many settings, and opioid maintenance treatment is evidence-based for the treatment of opioid use disorders. However, access to such treatment varies and is limited in many settings.

**Methods:**

The present study examines the longitudinal effects of a regional patient choice reform which substantially increased availability to opioid maintenance treatment in one Swedish county, starting from 2014. A previous follow-up, limited in time, indicated a possible effect on mortality from this intervention, demonstrating a lower increase in overdose deaths than in counties without this reform. The present study follows overdose deaths through 2021, and compares the intervention county to the remaining parts in the country, using death certificate statistics from the national causes of death register.

**Results:**

The present study does not demonstrate any significant difference in the development of overdose mortality in the county where this reform substantially expanded treatment access, compared to other counties in the country.

**Conclusions:**

The study underlines the importance to maintain extensive efforts against overdose deaths over and above the treatment of opioid use disorders, such as low-threshold provision of opioid antidotes or other interventions specifically addressing overdose risk behaviors.

**Supplementary Information:**

The online version contains supplementary material available at 10.1186/s13011-023-00577-4.

## Background

Opioid maintenance treatment (OMT) is a well-established and evidence-based treatment for opioid use disorders [1]. Sweden is a setting where OMT historically was limited and hard to access, but with a gradual increase starting in the 2000’s [2]. In Sweden, however, mortality for drug overdoses, primarily opioid overdoses, has increased and stabilized on high levels [3, 4]. This also includes a large increase in methadone-related overdoses, primarily outside of methadone treatment [5]. Likewise, the numbers of fatal poisonings with buprenorphine have increased [3, 6]. An overall impression of overdose deaths in recent years is that these continued to involve heroin metabolites (21%) as they did historically, but also involve a large number of cases whose fatal overdoses involved no heroin but instead methadone (43.5%) or buprenorphine (15.5%), and with a non-negligeable proportion of opioids typically used in pain management, such as oxycodone (6%) or fentanyl (15%). Also, sedatives, including benzodiazepines, were very common (84%) in these opioid poisonings [8]. Thus, overdose mortality rates have remained a substantial problem, also in comparison to most other comparable countries [3].

In 2014, a patient choice reform was implemented in the Skåne county in the southernmost part of Sweden. This reform led to a large increase in the number of treatment units, which compete on a market where their income depends on the number of patients admitted for OMT to the unit, and which are financed by the public authorities in the region. Overall, this has led to a substantial increase in the number of patients treated with OMT in this county [7]. A previous scientific paper assessed overdose mortality before and during the patient choice reform in the county. In that paper, it was reported that in the Skåne county, there was an ‘annual relative decrease in unintentional deaths in Skåne compared to the rest of Sweden following the onset of the reform’, and the relative risk was calculated to 0.90 (a 10% decrease) in comparison to the rest of the country [8]. Thus, although mortality from opioid poisoning (‘overdose’) increased over time during the study, and although no conclusion is specifically drawn from this analysis, the results of the data may suggest that the patient choice reform could have contributed to a smaller increase in overdose death than in the rest of the country. Thus, this comparative data suggests a favorable effect of the reform on overdose mortality.

However, the data reported above included a time period stretching from 2011 to 2017, and may require an update, especially given the continuing concern about the level of drug-related mortality in Sweden. For this reason, the analysis reported below aimed to continue the observation of how regional overdose death rates evolved in the county implementing the patient choice model, compared to the rest of the country.

## Methods

In order to facilitate comparison with the previously published paper on the same topic, we aimed to collect data for overdose deaths in Swedish adults (20–64 years of age) for the time period 2011–2021. The Cause of Death Register data is available since 1997, and we included data for all available years in order to represent the long-time trends visually.

### Data sources

We collected data from two publicly available sources. Data for causes of death was collected from the public database of statistics of the Swedish National Board of Health and Welfare. This data is registered in the Swedish Cause of Death Register and it comprises data from all deaths of people registered as living in Sweden, regardless of whether they were registered as living in Sweden or not. In the register, the ultimate cause of death, as determined by a medical doctor, is registered with codes from ICD-10. In the publicly available data, the number of people who died each year with a certain ICD-10 code group (individual ICD-10 codes are aggregated in meaningful groups) are reported, divisible by sex, age, and county. We thus included data for the number of individuals who had died each year with an ultimate cause of death coded as either X42 (accidental poisoning by and exposure to narcotics and hallucinogens), X44 (accidental poisoning by and exposure to other and unspecified drugs), or Y10-Y14 (poisoning with drugs with undetermined intent), within the age rage 20–64 years. Data was divided by county (Skåne county vs. all the other 20 counties in Sweden combined).

The other data source was the Total Population Register from Statistics Sweden. This is a register on all inhabitants of Sweden, publicly available for extraction of aggregate data by various variables. For this study, we collected data on the numbers of individuals registered as living in Sweden in each county for the years 1997–2021. We then summed the data for the 20 counties that were not Skåne in order to use the population data as an offset variable in the statistical analyses described below.

### Variables

The variables included from the register data were as follows: *overdose deaths*, *population*, *county* (Skåne vs. the other 20 counties in Sweden combined), and *year* (1997–2021, numbered). For purposes of statistical analysis, we included data only from 2011 and onwards.

We then created other variables based on the implementation of the intervention under study, i.e. the patient choice reform for OMT. This reform was gradually implemented in Skåne in 2014, so we excluded 2014 from the data for Skåne and designated 2011–2013 as pre-intervention and 2015–2021 as post-intervention. This dichotomous variable was labelled *intervention*. Because the effect of the intervention might be conceptualized as gradual rather than instantaneous, we created a new variable labelled *intervention slope* which was 0 for data points prior to 2014, and from 2015 to 2021 the data was 1 through 7.

The county variable can be used in order to assess the difference in means for Skåne vs. the rest of Sweden. We created interaction terms between county and the two intervention variables, to create the variables *county X intervention*, which has the value of 0 for all years prior to the intervention and 1 for all years post intervention for the data from Skåne county, and the value 0 for all years for the data from the rest of Sweden; and *county X intervention slope*, which differs from the previous variable only in that the data for the years following the intervention in the Skåne data has values from 1 to 7.

### Statistical methods

We analyzed the data as an interrupted time series using a Poisson regression framework, using identical statistical methods as in the previous paper [8] but with four more years of data. *Overdose deaths* was used as the dependent variable with *population* as an offset variable, allowing us to analyze the differences in death rates. *Year* was included as an independent variable in order to take into account the overall temporal trend in the data. It is clear from the data prior to the intervention date that there is no difference between Skåne county and the rest of Sweden in either mean or slope, so any variables that might reflect such a difference were omitted a priori.

For the remaining four possible independent variables, i.e. *intervention, intervention slope*, *county X intervention*, and *county X intervention slope*, we used two analysis strategies. In the previous paper by Andersson and colleagues [8], a model selection process was utilized, indicating that the best fitting model included the intervention slope and the interaction between county and intervention slope, and this model was selected for the main results. The first analysis strategy was to use the same model as was determined as best fitting in the previous paper, and this model will henceforth be referred to as *Model A*. The second analysis strategy was to repeat the same model selection process and select the model that showed the best fit.

The model selection process included all the combinations of the two variable sets below:

1. Either *intervention, intervention slope*, or none of them.
2. Either *county X intervention*, *county X intervention slope*, or none of them.

We thus created nine models which all included year and up to two of the other four eligible independent variables. The model fit was assessed by Akaike Information Criterion (AIC) and Bayesian Information Criterion (BIC), and the best fitting model was determined to be the model that included *year*, *intervention slope*, and *county X intervention mean*. This was the eight model of the nine models included in the model selection process, but is henceforth referred to as *Model B*.

The Poisson regression analyses performed in the model selection process are described in a supplementary table (table S1). The full data set is included in the supplementary material for full transparency (table S2). All analyses were performed using *R* 4.0.2 [9].

## Results

**Fig. 1:**
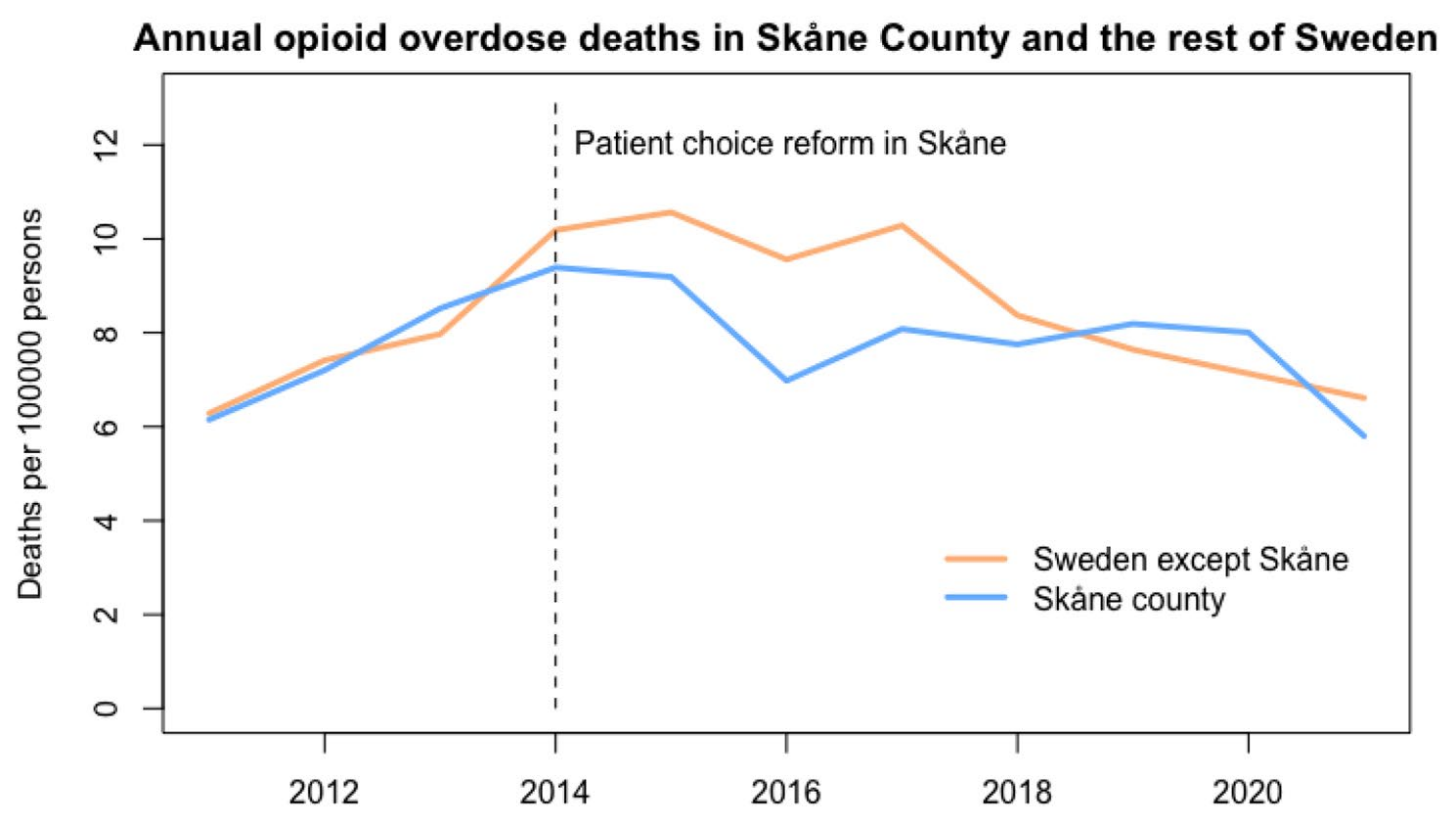
Annual opioid overdose deaths in Skåne County and the rest of Sweden

During the years 2011–2021, there was an average of 474 opioid overdose deaths per year (range 346 to 588) among people in the age range of 20–64 years in Sweden, corresponding to an average annual death rate of 8.3 per 100,000 individuals (range 6.3 to 10.4). The average annual death rates per 100,000 individuals were 7.8 in Skåne County and 8.0 in the rest of Sweden prior to the intervention date, and 7.7 in Skåne County and 8.6 in the rest of Sweden after the intervention date (Fig. 1).

The results from the two Poisson regression models are shown in Table 1. In both *Model A*, which was derived from the previous paper, and in *Model B*, which was identified as having the best model fit in the model selection process, the variables *Year* and *Intervention slope* were both significant at the p < .001 level with *Year* being higher than 1, indicating an increase by each year, and *Intervention slope* being negative, indicating a decrease by each year following the intervention date. In *Model A*, the rate ratio for *County X Intervention level* was 0.91 (95% confidence interval 0.83–1.01), indicating a decreased death rate following the intervention in Skåne compared to the rest of Sweden, though not statistically significant (p = .086). In *Model B*, the death rate per year for Skåne compared to the rest of Sweden, modelled with *County X Intervention slope*, was 0.99 (95% CI 0.96–1.01, p = .296).

**Table 1:** Results from the main Poisson regression models

	Model A		Model B	
Variables	RR	p	RR	p
Year	1.21 (1.17–1.25)	< 0.001	1.21 (1.16–1.25)	< 0.001
Intervention slope	0.78 (0.74–0.81)	< 0.001	0.78 (0.75–0.82)	< 0.001
County X intervention level	0.91 (0.83–1.01)	0.086		
County X intervention slope			0.99 (0.96–1.01)	0.296

## Discussion

The updated overdose mortality data reported in the present study, allowing a longer observation period than in the paper referred to above [8], concludes that it cannot be claimed that the patient choice reform for OMT in one Swedish county had affected overdose mortality in comparison to mortality in the rest of the country, where such a reform has not been implemented. In addition to concluding this, we call for further research and policy work aiming to address the highly concerning mortality rates seen in the present setting.

OMT remains one of the prioritized interventions in the management of opioid use disorders. For example, in the present setting, over time, time periods in OMT have been associated with a lower risk of overdose death than in periods out of OMT [10]. Thus, in addition to the international evidence of OMT [1], a generally favorable effect from OMT has also been suggested in the setting studied here. However, from the data available from the present county, it can hardly be concluded that the present type of OMT expansion, following a patient choice-oriented model with a large expansion in the number of treatment units, would decrease overdose mortality. It must instead be concluded that overdose mortality in the present setting remains high, and that further interventions – in addition to OMT – are needed to lower mortality, while also maintaining adequate access to evidence-based treatment. This may include overdose prevention programs [11], including the low-threshold distribution of the antidote naloxone, which have been shown to be promising [12] and can be considered over and above structured treatment of the opioid use disorder as such. Also, importantly, we argue that the findings from recent studies, such as the one from the present county, point to the importance of preventing benzodiazepine use in individuals who use illicit opioids, as the combination of these two substance groups was very common in opioid overdose victims [8].

### Electronic supplementary material

Below is the link to the electronic supplementary material.

Supplementary Material 1

## Data Availability

Original data used in the present study is included in supplementary Table 2 of the submission.

## List of abbreviations

AIC: Akaike Information Criterion
BIC: Bayesian Information Criterion
ICD-10: International Classification of Diseases (10th edition)
OMT: Opioid maintenance treatment
