# Supplementary material for: No obvious effect on mortality from a patient choice reform expanding access to opioid disorder treatment – results from a natural experiment of policy change in Sweden
Source: Subst Abuse Treat Prev Policy. 2023 Nov 6;18:64. doi: 10.1186/s13011-023-00577-4 (PMC10629127; doi:10.1186/s13011-023-00577-4)
Supplement: Supplementary file 1 — Supplementary Material 1 [file 13011_2023_577_MOESM1_ESM.docx]

| **Supplementary table S1. Model selection criteria for Poisson regression models.** | | |  |
| --- | --- | --- | --- |
| **Model** | **Variables included** | **AIC** | **BIC** |
| Model 1 | Year | 302.7 | 304.8 |
| Model 2 | Year + Intervention mean | 268.6 | 271.7 |
| Model 3 | Year + Intervention slope | 179.5 | 182.6 |
| Model 4 | Year + County X Intervention mean | 302.9 | 306.1 |
| Model 5 | Year + County X Intervention slope | 301.4 | 304.5 |
| Model 6 | Year + Intervention mean + County X Intervention mean | 266.3 | 270.4 |
| Model 7 | Year + Intervention mean + County X Intervention slope | 267.7 | 271.9 |
| Model 8 | Year + Intervention slope + County X Intervention mean | 178.5 | 182.7 |
| Model 9 | Year + Intervention Slope + County X Intervention slope | 180.4 | 184.6 |

**Supplementary table S2. Full data set used in the present study.**

| **Year** | **Deaths** | **Population** | **Time** | **County group** | **Intervention level** | **Intervention slope** | **County X Intervention level** | **County X Intervention slope** |
| --- | --- | --- | --- | --- | --- | --- | --- | --- |
| 2011 | 301 | 4789579 | 1 | 0 | 0 | 0 | 0 | 0 |
| 2012 | 357 | 4815167 | 2 | 0 | 0 | 0 | 0 | 0 |
| 2013 | 386 | 4843330 | 3 | 0 | 0 | 0 | 0 | 0 |
| 2014 | 497 | 4879618 | 4 | 0 | 0 | 0 | 0 | 0 |
| 2015 | 519 | 4913499 | 5 | 0 | 1 | 1 | 0 | 0 |
| 2016 | 475 | 4968163 | 6 | 0 | 1 | 2 | 0 | 0 |
| 2017 | 515 | 5007303 | 7 | 0 | 1 | 3 | 0 | 0 |
| 2018 | 422 | 5041214 | 8 | 0 | 1 | 4 | 0 | 0 |
| 2019 | 388 | 5077153 | 9 | 0 | 1 | 5 | 0 | 0 |
| 2020 | 363 | 5090056 | 10 | 0 | 1 | 6 | 0 | 0 |
| 2021 | 338 | 5110476 | 11 | 0 | 1 | 7 | 0 | 0 |
| 2011 | 45 | 731962 | 1 | 1 | 0 | 0 | 0 | 0 |
| 2012 | 53 | 735957 | 2 | 1 | 0 | 0 | 0 | 0 |
| 2013 | 63 | 739599 | 3 | 1 | 0 | 0 | 0 | 0 |
| 2014 | 70 | 745584 | 4 | 1 | 0 | 0 | 0 | 0 |
| 2015 | 69 | 750676 | 5 | 1 | 1 | 1 | 1 | 1 |
| 2016 | 53 | 759527 | 6 | 1 | 1 | 2 | 1 | 2 |
| 2017 | 62 | 767278 | 7 | 1 | 1 | 3 | 1 | 3 |
| 2018 | 60 | 774018 | 8 | 1 | 1 | 4 | 1 | 4 |
| 2019 | 64 | 781339 | 9 | 1 | 1 | 5 | 1 | 5 |
| 2020 | 63 | 786779 | 10 | 1 | 1 | 6 | 1 | 6 |
| 2021 | 46 | 793010 | 11 | 1 | 1 | 7 | 1 | 7 |
